# Genomic Tackling of Human Satellite DNA: Breaking Barriers through Time

**DOI:** 10.3390/ijms22094707

**Published:** 2021-04-29

**Authors:** Mariana Lopes, Sandra Louzada, Margarida Gama-Carvalho, Raquel Chaves

**Affiliations:** 1 Laboratory of Cytogenomics and Animal Genomics (CAG), Department of Genetics and Biotechnology (DGB), University of Trás-os-Montes and Alto Douro (UTAD), 5000-801 Vila Real, Portugal; lopesfmariana@gmail.com (M.L.); slouzada@utad.pt (S.L.); 2 Biosystems and Integrative Sciences Institute (BioISI), Faculty of Sciences, University of Lisbon, 1749-016 Lisbon, Portugal; mhcarvalho@fc.ul.pt

**Keywords:** satellite DNA families, satellite DNA characterization, variability, genomics, technique interdependency

## Abstract

(Peri)centromeric repetitive sequences and, more specifically, satellite DNA (satDNA) sequences, constitute a major human genomic component. SatDNA sequences can vary on a large number of features, including nucleotide composition, complexity, and abundance. Several satDNA families have been identified and characterized in the human genome through time, albeit at different speeds. Human satDNA families present a high degree of sub-variability, leading to the definition of various subfamilies with different organization and clustered localization. Evolution of satDNA analysis has enabled the progressive characterization of satDNA features. Despite recent advances in the sequencing of centromeric arrays, comprehensive genomic studies to assess their variability are still required to provide accurate and proportional representation of satDNA (peri)centromeric/acrocentric short arm sequences. Approaches combining multiple techniques have been successfully applied and seem to be the path to follow for generating integrated knowledge in the promising field of human satDNA biology.

## 1. Introduction

Back in the 1960s, Britten and Kohne revealed the high abundance of repetitive sequences in eukaryotic genomes [1], opening up a new field of research. Notwithstanding, the biological importance of these repeated sequences was perpetually neglected for many years to come, as the repetitive portion of the genome was often dismissed as non-functional (simply ‘‘junk DNA’’) [2]. This non-functional term itself has long been troublesome, as it is argued that even in the early days some researchers stated the likelihood of a functionality for repetitive DNA [3].

Soon, the need to classify repetitive DNA sequences arose, first in a major classification related to repeat number and subsequently in group classifications according to their organization (arrays of tandem repeats or interspersed sequences) [4]. Tandem repeats are characterized by the adjacent alignment of sequence units in a hierarchically organized manner, while interspersed repeats have a scattered, multi-locus distribution across the genome [5,6].

The term “heterochromatin” was coined in 1928 [7], in parallel with the assessment of its distinctive state of constant compaction. As a consequence, heterochromatic genomic regions were portrayed as silent and inert, an assumption that became inevitably associated with repetitive sequences, being the major heterochromatic component [8]. In the wake of the disclosure of the repetitive fraction of the genome, a new class of tandemly repeated DNA sequences was first revealed in 1961 [9,10] and later identified in the 1970s as the constituent of satellite peaks in cesium chloride density gradients. Essentially, satellite bands were differentiated from the remaining genomic DNA by their A/T content [11]. The name satellite DNA (satDNA) was here to stay [12] and has been used ever since as a broader term for tandem repeats [13]. Fundamentally, satDNAs make up the eukaryotic centromeric and pericentromeric genomic regions [14], even though they can also be located at subtelomeric sites or even at interstitial regions [15,16,17]. SatDNA sequences can be distinguished by a multitude of dissimilar features, like nucleotide sequence composition, complexity, and abundance, although their major shared characteristics cannot be dismissed: the capacity to form heterochromatic regions and the intrinsic propensity to form long tandemly organized arrays [17]. The apparent unlikelihood of a discernible role ascribed to repetitive sequences (and therefore to satellite DNA) did not prevent the continuous emergence of a variety of studies, essentially focusing on investigating these sequences in terms of their associated functions [5,11,14,18,19,20,21,22,23,24,25]. Thus, the idea of a potential function was parsimoniously considered: the common presence of genomic repetitive sequences could have some underlying meaning [17].

We now know that a variety of vital cellular processes is influenced by satDNA arrays: cell cycle, gene expression, or even genome stability [26]. Being a constitutive element of key structures such as centromeres or telomeres, satDNA has been phenotypically associated with chromosome and cell function in multiple species, specifically in humans [27,28], in which several satDNA families have been progressively identified and characterized, albeit not at the same pace.

## 2. Human Satellite DNA Families

Going back to the 1960s, the discovery and classification of three clearly distinguishable human genomic DNA fractions in CsSO4 gradients established the identity of the corresponding classical satellite DNAs I, II, and III. More precisely, a set of repetitive sequences with analogous buoyant densities was found to compose each gradient fraction [29]. These DNA fractions presented a characteristic inter-sequence heterogeneity, which led to a new classification in 1987, as a prime family of simple repeats was identified for each fraction [30]. The three families were described as satellite DNA families I, II, and III [29] and were first reported to be present in all acrocentric chromosomes, as well as in chromosomes 3 and 4 [31]. Additionally, the centromeric alpha (α) satellite DNA family was also identified and described, soon becoming the most intensively studied human satDNA sequence. Later on, gamma (γ) and beta (β) satellites were likewise found among the diverse families of human satellite DNAs [32].

Through processes of amplification and homogenization, some satDNA monomers have the ability to form Higher-Order-Repeats (HORs) units [17]. In fact, it has been demonstrated that HOR structure can influence regulated functions (like gene expression [33,34] and replication efficiency [35,36,37]) and have a significant role in individual/populational diversity [38,39].

Today, the larger satellite regions of classical heterochromatin in the human genome (essentially present in chromosomes 1, 9, and 16 and acrocentric short arms) [40] remain poorly understood. The known complexity of these regions and associated lack of knowledge cause a significant void: the lack of an integrated portfolio describing different subfamilies, possibly facilitating variability studies within the same satellite family [41]. For instance, the heterochromatic band of chromosomes 1, 9, and 16 represents a source of human variation [41], given that assessable polymorphisms are found between individuals [42,43,44]. The Y satDNA repeats represent an additional example of how satDNA may constitute a valuable tool to study human variation, given that the frequency of satellite variants can fluctuate greatly between individuals [40]. Indeed, variations within satellite arrays have been shown to influence the overall size of the Y chromosome across human populations [41].

Twenty years ago, different satellite subfamilies were already recognized to have different features. In spite of the constant repeat unit size, sub-variability within the same satDNA family was reported [45], feasibly explained by a different organization and, therefore, different clustered localization [46]. The differential chromosomal location of satellite subfamilies has been mostly shown by clonal-based fluorescent in situ hybridization (FISH) studies [30,31,45,47,48,49,50,51]. In accordance with these early studies, it was demonstrated that different subfamilies do not correspond to different parts of the same array but instead coincide with genomically separated subgroups [41]. This type of genomic analysis is essential, since satellite-associated information gaps are responsible for uncertainty issues regarding the exact number of satDNA families/subfamilies (e.g., families reported as different might be related or different subfamilies may compose the same satDNA family) [52]. In the next sections, we introduce the present-day understanding about the most significantly described human satDNA (sub)families.

### 2.1. α Satellite DNA

Initially, α satellite DNA (*αSAT*) was isolated from a highly repetitive fraction present in the African green monkey genome [53]. Subsequently, α satellite repeats were shown to be present in all human centromeres and to be composed of tandem repeats of an AT-rich 171 bp-long monomer [54,55,56,57]. Alphoid monomers can form HORs composed of nmer repeats (being *n* the number of monomers) or be organized in a non-HOR manner as simple monomeric repeats [28]. HORs can be formed by 2 to 34 monomers [28,56,58,59]. Some monomers within α satellite HORs have a 17 bp sequence motif called the Centromere Protein B (CENP-B) box because of the ability of CENP-B to recognize and bind to these regions [60]. The CENP-B box location is structurally related to the chromosome-specific HOR array (varying accordingly) [5]. Moreover, the CENP-B box is present with high degree of conservation in other mammal genomes [61]. Studies show that an active CENP-B box is required for de novo centromere assembly in humans, acting in the recruitment of the Centromere Protein A (CENP-A) and stabilization of the Centromere Protein C (CENP-C) [62], both related to an active kinetochore and proper chromosome segregation, which might explain its retention in different genomes. Each human chromosome contains one or more exclusive α HOR array, except for chromosomes 13/21 and 14/22, which share the same HOR array [46,50,63]. Regarding acrocentric chromosomes, a variety of α satellite subfamilies can be found in the vicinity of the centromere: pTRA-1, pTRA-2, pTRA-4, and pTRA-7, all of them present in chromosomes 13, 14, and 21 [46,64]. These subfamilies are part of a catalog of 28 clone-isolated α subfamilies from all human chromosomes [64], although, presently, an accurate genomic analysis is still required to avoid redundant classifications.

α satellite soon became the model for the hierarchical HOR organization [29]. Alphoid sequences are deeply related to proper cell division (being the foundation for kinetochore formation); the occurrence of active centromeres; and, therefore, centromere identity. It is possible to distinguish human centromeres based on their α HOR specificity-conferring composition, namely, by the number and order of monomers (that share 50–70% of identity) [65]. By defining α monomer consensus sequences, it is possible to discern five suprachromosomal groups or subfamilies, based on the possible monomer combinations [65,66] (reviewed in [5,66]). The main suprachromosomal subfamilies (SF1-3) correspond to the kinetochore formation region and are associated with centromere functionality [67,68]. Hybridization studies performed at high stringency allow the mapping of individual HORs to specific chromosomes [56] because of sequence polymorphisms found between them [5]. At low stringency, subsets of HOR arrays co-hybridize, allowing one to study how suprachromosomal subfamilies relate to each other [58,69]. Beyond the occurrence of α HORs, α monomeric repeats are present in transitory, array-adjacent pericentromeric regions, feasibly evolving non-homogeneously from homogenous HORs [59,70]. The relative mutation rate of centromeric α satellite sequences (accelerated comparing to unique genomic portions) lines up with a layered and symmetric evolution in the following direction: active HOR repeats-ancient HOR repeats-monomeric repeats [71]. In fact, closeness to the functional core centromere is a determining factor for HOR homogenization, as distant monomers are considered older, more variable, and a trace of centromere primate evolution [72]. Therefore, HOR array chromosome specificity results from intrachromosomal homogenization [13].

### 2.2. Satellite DNA I

SatDNA I (*SATI*) is distinguished by the presence of 42 bp repeats, consecutively arranged in units of 2 types, A (17 bp) and B (25 bp) repeat units [29], which can tandemly organize in ABABA constructs [30,73]. *SATI* repeats can form HORs of 2.97 Kb [74]. The amplification of these sequence arrays arranged in a head-to-tail fashion resulted in the current complexity of the *SATI* DNA family [73]. *SATI* is the most AT-rich fraction of the human genome, being also the least abundant classical satellite [51]. This classical satellite was first described using a probe (pTRI-6) that hybridizes with all acrocentric chromosomes at low stringency and only with chromosomes 13 and 21 at high stringency [46,74]. Until this day, pTRI-6 remains the only sequence described as a *SATI* subfamily. In 1986, experiments with the restriction enzyme RsaI allowed for the detection of the ABABA construct [30]. In this study, the A-B 42 bp form was considered predominant, although a possible second form (B-B dimers) was also observed. Later, Meyne et al. determined that B-B repeats hybridize with chromosome 3 and acrocentric chromosomes. The predominant ABABA construct showed an analogous chromosomal location to that of the pTRI-6 subfamily (chromosomes 3 and 4 and acrocentric). Acrocentric hybridization signals could be found in two locations: proximal pericentromeric and more distal short arm regions. The authors highlighted the need for high-resolution molecular studies for variant analysis [73], a requirement that remains pressing a quarter a decade later.

### 2.3. Satellite DNA II/III

SatDNA II (*SATII*) associates with a poorly conserved repeat unit (ATTCC), and satDNA III (*SATIII*) was shown to be composed of pentameric repeats of the same motif (well-conserved and interspersed with a specific 10 bp sequence) [29,75]. The inconsistent arrangement of satellite II/III in complex repeats (as opposed to tandem repeats) has led to a poor characterization of these satellite families [41]. *SATII* and *SATIII* probably arose from the same pentameric repeat [30], yet today these sequences locate to different genomic regions [41,48].

*SATII* repeats were initially reported to predominantly locate at chromosome 1 [45,48] and chromosome 16 [76,77]. In particular, the chromosome 1 *SATII* array represents a chromosome-specific 1.77 kb unit [48]. To a smaller extent, *SATII* was also found in pericentromeric regions of chromosomes 2 and 10 [45]. In 2014, three different *SATII* subfamilies were analyzed, presenting different sequence composition and genomic location [41]. Today, the chromosomal location of *SATII* family is more broadly recognized, supported by increasing genomic and bioinformatic studies.

*SATIII* was localized to chromosomes 1, 9, and Y [76,77,78], as well as to acrocentric short arms [79]. *SATIII* repeats have likewise been progressively found in additional chromosomal locations (e.g., chromosomes 5, 10, 17, and 20) [29,45]. *SATIII* presence in the Y chromosome long arm is distinguished by a male-specific 3.6 Kb repeat unit [78,80,81]. With respect to *SATIII* acrocentric repeats, eleven different subfamilies have been identified and characterized: pTRS-47 [82], pTRS-63 [83], pTR9-s3 [31], pTRS-2 [46], pE-1, pE-2, pR-1, pR-2, pR-4, pK-1, and pW-1 [84]. The pTRS-47 and pTRS-63 subfamilies, located at chromosomes 14/22 and 14, respectively, seem to be particularly significant in a clinical context for their involvement in the breakpoint of human Robertsonian translocations [85]. Interestingly, computational clustering analysis of human sequences was able to identify a total of eleven *SATIII* subfamilies [41]. Although the number of identified subfamilies correlates with previous clone hybridization studies on acrocentric chromosomes, predicted chromosomal locations do not seem to match (not all identified subfamilies locate to acrocentric chromosomes). A comprehensive sequence analysis is essential since different sequence composition and physical locations (observed in both approaches) point to the existence of a higher number of *SATIII* subfamilies. Gaps in our understanding of *SATII*/*SATIII* repeats are strongly associated with limiting bioinformatic/sequencing approaches, due to their short irregular nature [41], as well as close sequence relation.

### 2.4. β Satellite DNA

β satDNA (*βSAT)* was initially named as Sau3A satDNA family [86] and effectively termed β satellite in 1989 [87]. β satellite repeats consist of tandem arrays of a 68 bp monomer organized in multimeric HORs, described to be present in all acrocentric chromosomes and chromosomes 1, 3, 9, 19, and Y [47,79,86,87,88,89], predominantly in pericentromeric regions [90]. Indeed, β satellite was distinguished in two different types of HORs (pB3 and pB4), composed of non-overlapping arrays with distinct genomic locations. pB3 is specifically localized in chromosome 9, and its representation is equivalent to 50–100 times per haploid genome. The second type of HOR, pB4, is 5 times more represented per haploid genome and is located in acrocentric chromosomes, where β satellite was found early on to map distally and proximally to rDNA [87]. Recently, β satellite was identified to be present in multiple eukaryotic taxa and to be the object of horizontal transfer (HT) events, contradicting previous claims of its exclusive presence in primates [91].

### 2.5. γ Satellite DNA

Originally, γ satDNA (*γSAT*) was isolated from a chromosome 8 specific clone [92]. Later on, another γ subfamily was described in chromosome X [93]. Known γ satellite subfamilies (GSAT, GSATX, and GSATII, with ~60% identity) are GC-rich tandem pericentromeric repeats of a vastly diverged 220 bp monomer and have been identified in all human chromosomes [40,94] usually forming clusters of 2–10 kb [92]. Kim et al. [94] proposed that γ satellite repeats may possibly work as barriers for heterochromatin expansion to chromosomal arms, being functionally similar to genomic insulators. This thesis emerged in accordance with previous statements regarding the existence of structural and functional constrains related to γ satellite [29].

Regardless of the common satDNA composition, centromeric and pericentromeric chromatin are structurally different, essentially because centromeres are epigenetically compatible with kinetochore assembly and chromosome segregation, while pericentromeric regions have a typical heterochromatic behavior [26]. Thus, the ubiquitous centromeric presence of α satellite sequences is contrasted by the nature of pericentromeric satellite families that clearly behave in a more non-homogenous manner [23,29,55], frequently leading to incongruences about their overall existence and location in the human genome [95]. Human centromeres are not only composed of satellite sequences, but also contain mobile elements, including LINEs and SINEs (Long/Small Interspersed Nuclear Elements), already described both in HOR arrays and monomeric repeats [13,95]. Hence, the centromeric region of human chromosomes is mostly composed of α HORs, eventually punctuated by transposable elements (TEs) [96,97], and progressively replaced by pericentromeric satellite families (classical satellites and β/γ satellites) [23]. Table 1 presents a summary of the available information about human satellite families.

## 3. Satellite DNA: Repetitively Challenging

The heterogeneity of known human satellite families constitutes evidence as to why the satDNA field has struggled with terms and definitions. If we take repeat unit size as an example, defining classical satellite sequences as microsatellites (≤10 bp) or minisatellites (between 10 and 100 bp) [6,100,101] is straightforward reasoning. However, these sequences tandemly organize in long arrays in heterochromatic regions, having a known satellite-like behavior. So, to categorize sequences with smaller monomer sizes as satDNAs (classically with longer repeat unit sizes) is also plausible [52,102]. The uncertainty found in human satDNA features (such as identification, existence, location, and others) is caused by the complex assembly of the (peri)centromeric regions [95,103]. The organization of *αSAT* (the most studied human satellite) in the human centromere clearly shows this intricacy: each HOR array is composed of the same monomer set; however, HOR composition is responsible for chromosome specificity, and HOR repeat number is distinguishable between individuals and even between homologous chromosomes of the same individual [66].

The vast structure of the human centromere has been paradoxically suppressed since the apparent completion of the human genome sequence map by the human genome project (HGP) consortium in 2003 [104], which in reality excluded 10% (or more) of genomic elements, specifically large portions of repetitive (peri)centromeric sequences [103] and acrocentric short arm sequences [105]. Today, the human genome reference GRCh38 still contains 161 Mbp of undetermined sequences (up to 5% of the genome) [106]. The latter fact is striking given that, using computational tools like the Tandem Repeats Finder [107] and Repeat-Masker, it was possible to generate evidence suggesting that ~1 million tandemly repeated sequences exist in the human genome (according to the UCSC annotation) [108,109].

Highly repetitive satellite DNA undoubtedly represents a major gap in human genome assemblies, significantly contributing to the lack of high-resolution sequencing studies in the field of centromere genomics, whose characterization has been substantially hindered by the repetitive nature of satDNA [26,110,111]. The availability of computer software algorithms for sequence analysis has been highly restricted to methods excluding repetitive sequences and disregarding their annotation [6,97,111]. Reads repetitive in nature (i.e., mapping to multiple locations) are generally overlooked. These problems cause misalignments and misassemblies [112] with a high number of contigs, assigning untraceable genomic positions to the analyzed repeats [113]. It is a known fact that satellite repeats are significantly represented in assembly pools, but the precise determination of their location in linear stretches within centromeric regions becomes unfeasible [5]. Theoretically, the correct placement of centromeric repeats in a linear assembly requires the availability of distinctive sequence information [114], which cannot be due to sequencing errors and is often not found in sequencing reads from homogenized satellite arrays [115,116,117], at least until recently.

An improved contiguity of the human reference genome is of crucial importance in the case of repetitive satellite sequences. Compared to the previous assembly, the GRCh38 human reference genome provides for the first time a representation of centromeric sequences, with the former gaps replaced by the insertion of millions of bases of chromosome-specific α satellite repeats identified from sequencing reads, modeling each centromere. Although this inclusion is expected to have a positive impact on the mapping and assembly of sequencing reads [118], the sole inclusion of *αSAT* sequences [95] undermines accurate and proportional representation of repetitive sequences, biasing data interpretation [119]. If we start from an incomplete and collapsed set of satellite data, we certainly ignore uncharted, possibly relevant information, especially information related to pericentromeric satDNA families. Acrocentric chromosomes, often lacking unique markers and sequence heterogeneity, are especially affected by this obstacle [114]. Within the same HOR array, HORs have little variability. So, centromere sequence assembly has to rely on monomer rearrangements [96,120,121] causing single nucleotide variants or large structural variants [122,123]. Hence, to the challenge of sequencing a single HOR array, acrocentric chromosomes add the difficulty of HOR array sharing and sequence similarities concerning pericentromeric subfamilies.

Recently, a group of scientists initiated the telomere-to-telomere (T2T) consortium with the aim of produce high-quality, end-to-end assemblies for every human chromosome, sharing all the generated data with the scientific community. This consortium is settled to fix the remaining gaps in the human genome (particularly the sections composed by highly repetitive DNA, typically difficult to include in cloning, sequencing, or assembling processes [124]) by combining different sequencing technologies and bioinformatic tools (progress detailed in the Section 4.3).

## 4. Satellite DNA Analysis: The Promise behind Long Reads and Technique Interdependency

### 4.1. Sequencing Methodologies over Time

DNA sequencing technologies were developed to solve the pressing need of efficiently identifying the order of monomers in the largest biological molecule. The evolution path of sequencing methods was forged by Sanger sequencing in the 1970s; it was firstly unmanageable in terms of reproducibility but continuously developed and was soon seen as a standard for many years to come [125]. The dideoxy Sanger method allowed the obtention of the first sequenced genome (bacteriophage Phi-X174) [126] and many years later, was behind the first draft of the human genome [127,128]. In a prior time when Sanger sequencing was in development, the use of polyacrylamide gels was slow, laborious, and simply not compatible with larger, complex genomes (the human genome appeared unachievable) [129]. So, and thanks to their genomic abundance, repetitive sequences were an early present obstacle when sequencing the human genome. The development of capillary electrophoresis and fluorescent labelling allowed the development of highly parallel sequencing in automated sequencing machines [130,131,132,133], turning the first sequence of the human genome a visible reality. In 2003, the first sequence of the human genome was achieved by the International Human Genome Sequencing Consortium (IHGSC) and the private biotechnology company Celera genomics, using the same method for DNA sequencing but following different approaches. As the Sanger methodology generates sequence information below one kb (kilobase) in length, the fast determination of long fragments required the development of approaches based on the ‘‘shotgun sequencing’’ of cloned segments derived from randomly fragmented large molecular weight DNA, followed by sequence assembly into in silico contigs [134,135]. With the first map of the human genome, the need to efficiently tackle human genetic diversity laid the foundations for the appearance of new sequencing methods capable of addressing such issues. Next-generation sequencing (NGS) emerged, supporting high-throughput and cost-effective analysis, and considerably improving the sequencing process [136,137,138]. Throughout the years, the introduction of high-performance platforms has allowed the easy and low-cost obtention of a very high number of short reads and a novel understanding of genome complexity [139]. Despite the benefits, the limitations of NGS (or third generation sequencing) spurred the development of alternative technologies in order to reach the technological highpoint of sequencing with reads great in length, accuracy, and the use of native DNA [138,140].

Long-read technologies, like PacBio (Pacific Biosciences) or Oxford Nanopore Sequencing, can surpass some limitations of short reads, such as the profiling of tandem repetitive sequences [141]. Despite enabling highly accurate genotyping in non-repetitive genomic regions, technologies like short-read Illumina sequencing are not able to provide contiguous de novo genome assemblies, limiting the reconstruction of long stretches of repetitive sequences [142]. By turn, sequencing methods that produce longer read lengths have been shown to support a more accurate assessment of the size of repeated monomers in satellite sequences [143]. With clear chemical and functional differences, the two predominant long-read technologies—henceforth termed PacBio and Nanopore sequencing—have great potential in the study of previously unreachable (peri)centromeric and acrocentric short arm regions s [144]. The different approaches used by each technology affect read length, accuracy, and throughput, establishing distinct limiting factors. For example, the sequencing-by-synthesis principle used by PacBio, often called single-molecule real-time (SMRT) sequencing, has a higher accuracy but read lengths that are limited by the lifespan of the polymerase [145], constantly incorporating fluorescently labelled deoxynucleoside triphosphates [144]. In contrast, for Nanopore sequencing, read length limits essentially depend on the ability to obtain unfragmented high-molecular weight (HMW) DNA, whereas accuracy is dependent on the ability of base-calling algorithms to deconvolute the ionic current fluctuations established by the passage of molecules through a pore into a precise identification of the order of nucleotides [142,145]. The current read length record using R10.3 nanopore flow cells stands at 4.3 Mb, while the most recent analysis algorithm Bonito CRF is consistently supporting single read accuracies of 98% and SNP accuracies of 99.92%, comparable to short read accuracy [146]. In turn, PacBio counts with a level of accuracy of 99.8% [147], thanks to the latest circular consensus sequencing (CCS) technology [148], which produces the so-called high-fidelity (Hi-Fi) reads (longer than 10 kb) [144]. Both PacBio and Nanopore sequencing offer the opportunity to address long tandem repeats by analyzing their length, nucleotide composition, and nucleotide modifications. Nanopore Sequencing is particularly promising given its polymerase-free chemistry and related ability to tackle extreme GC content [149], while PacBio offers the increased accuracy of HiFi reads, specifically interesting for assembling centromeric repeats [150,151].

Given that a wide variety of satellite repeats can be transcribed in noncoding RNAs (ncRNAs), RNA sequencing (RNA-seq) has also become a part of satellite DNA research. Satellite DNA expression may depend on time, tissue, developmental state, or stress conditions [18,20,26], clearly making RNA-seq studies noteworthy and subject to constant evolution. In this matter, it is important to emphasize on the different methodological constrains of analyzing small or long ncRNAs. Long ncRNAs (lncRNAs) represent transcripts longer than 200 nucleotides, and contrarily to small ncRNAs, the great majority of their functions remain poorly understood [152,153]. The challenging nature of lncRNAs is essentially related to their features (for example, low abundance, structure conservation rather than sequence conservation, or even inconsistent polyadenylation) [152]. These differences are pertinent, given that satellite DNA transcripts are variable in size (from small to long ncRNAs) [20,154]. RNA-seq with short-read technologies traditionally relies on cDNA synthesis, reduced read length, and high sequencing throughput. On the other hand, newer long-read technologies, through long-read RNA-seq or direct RNA sequencing (dRNA-seq), are allowing one to address several questions related to RNA characterization. SatDNA transcripts that have distinctive features like length, strand specificity, nucleotide modifications, and polyadenylation [26]. The possibility of directly sequencing RNA (brought by Nanopore sequencing) [155,156] has removed the need for cDNA conversion or PCR amplification, significantly removing associated bias [157,158] and supporting the analysis of epigenetic modifications and 3′ poly(A) tails [159]. However, Nanopore sequencing of RNA molecules associates with higher error rates than DNA sequencing [158,160]. In this regard, PacBio (despite not allowing dRNA-seq) can face the problem of high error rates by increasing read coverage with consensus circular sequencing (CCS) [157].

### 4.2. Technique Interdependency

While assembling a genome, specific satellite-associated gaps arise due to the organization in HOR tandem arrays, causing read “pile-ups” during the overlap mapping stage that cannot be resolved [161]. With long-read sequencing, analysis of whole sections of repetitive DNA becomes more accessible, including HOR structure, TE interruption [162], or the accurate determination of monomer size [143]. The overall centromeric structure is finally within reach in a variety of circumstances.

**Figure 1:**
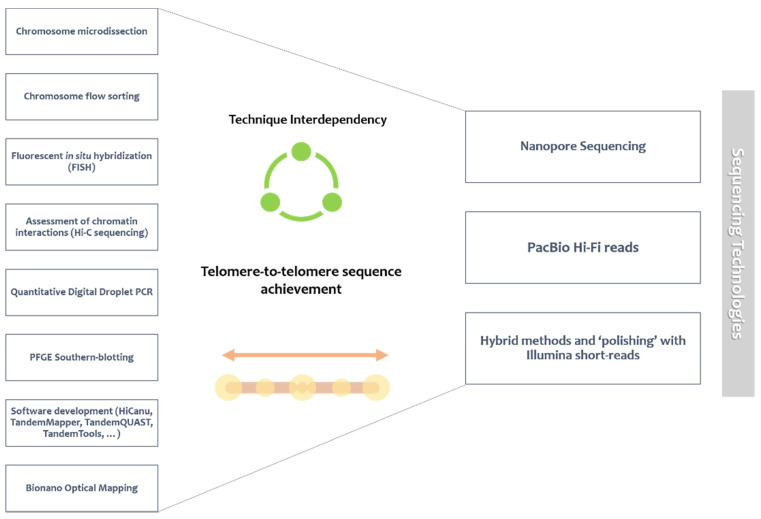
Telomere-to-telomere (T2T) assembly of human chromosomes relied on several different techniques. This combined approach was indispensable for closing the remaining gaps found in early assemblies (mostly related with telomeric, centromeric, and other interstitial regions like segmental duplications). The use of both PacBio and Nanopore sequencing, together with “polishing” methods, was applied as a sequencing strategy. The methodologies presented in the left allowed improve sequence mapping and assembly.

Nevertheless, the picture did not immediately present itself as all rosy for long-read technologies, as improved read length was often accompanied by increased error rate. Presently, the attempt to overcome this association is based on high-quality consensus and improved read coverage [114,163], which can still be improved by additional strategies for de novo genome assembly, especially in repetitive regions. This is where error correction enters, using non-hybrid or hybrid methods, in a process generally termed ‘‘polishing’’ (Figure 1). Non-hybrid correction is solely based on long reads; hybrid methods rely on the accuracy of supplementary short-read information [145]. The latter is usually applied in the context of a long-read-generated high-level genome assembly with repeat complexity, which is subsequently polished with short-read data for high-quality resolution (though the opposite is also applicable) [163,164,165,166].

Mapping strategies and cytogenetic approaches can also be applied to aid in the assembly process (Figure 1) [147], experimentally validating array structure [114]. Cytogenetic methodologies include optical mapping by Bionano, which uses restriction enzymes and fluorescent labels to obtain linearized genomic optical maps [147,167]; andfibre-FISH, where fluorescently labelled probes hybridize to stretched DNA [110,147,168]. Optical mapping, for instance, has been used in improving contig size or in scaffolding [169,170]. Additionally, PFGE Southern-blotting, making use of specific satellite array probes [122], and combined with other methods like quantitative digital droplet PCR [120], can help discover HOR array copy number and structure by comparing the resulting data with long-read assembly information [114,120]. Polishing methods and mapping-assisted assembly are co-dependent and intrinsically related. The obtainment of higher error rates with long-read sequencing greatly hampers precise sequence annotation [171], limiting the purpose of scaffolding methods (like optical mapping), which rely on base accuracy [172] (possibly guaranteed by polishing strategies) [173].

To sum up, genome sequencing projects can be greatly facilitated and provide integrated knowledge if combined with other fields of genome biology such as cytogenetics and cellular biology [174]. In addition to the already mentioned Bionano and fibre-FISH, several cytogenetic approaches have been used in interdependency with “orthodox” genomic studies (a changing concept, since genomic studies are progressively integrating research from all fields). These cytogenetic methodologies include chromosome microdissection [175,176,177], chromosome flow sorting [178], and assessment of chromatin interactions (e.g., Hi-C sequencing) [179,180]. Technologies like chromosome flow sorting, chromosome microdissection, and even magnetic bead capture offer an inventive, technical tackling of complex repetitive genomic regions, through the ability to isolate specific chromosomes/chromosomal regions [181,182]. Hence, this targeted capture can be applied to the task of sequencing a chromosome for which there is limited sequence information [181], which can be particularly interesting in the case of (peri)centromeric/acrocentric short arm sequences, given the extensive degree of sequence sharing between different chromosomes.

Cytogenetically-assisted chromosome-level assemblies undoubtedly offer more insightfulness than a disarray of fragmented contigs/scaffolds. To fully understand a genome, it is also necessary to recognize genome architecture and chromosome function. In order to improve genome assembly, all the above-mentioned technologies deeply depend on the development of adequate bioinformatic pipelines and software tools, which progressively need to adapt to new data forms. The concept of technique interdependency intricates algorithm development since it demands large data compacting structures, flexibility, and consequent speed-increased analysis [183]. Recently, a set of long-read tools has been put together to evaluate state-of-art software possibilities and identify missing useful pipelines with development potential (long-read-tools.org) [145]. Thus, the evolution of genome sequencing and mapping technologies must be supported by innovative bioinformatic tools with ability to overcome the computational challenge of combined data.

### 4.3. The Achieved Vs. the Achievable

**Figure 2:**
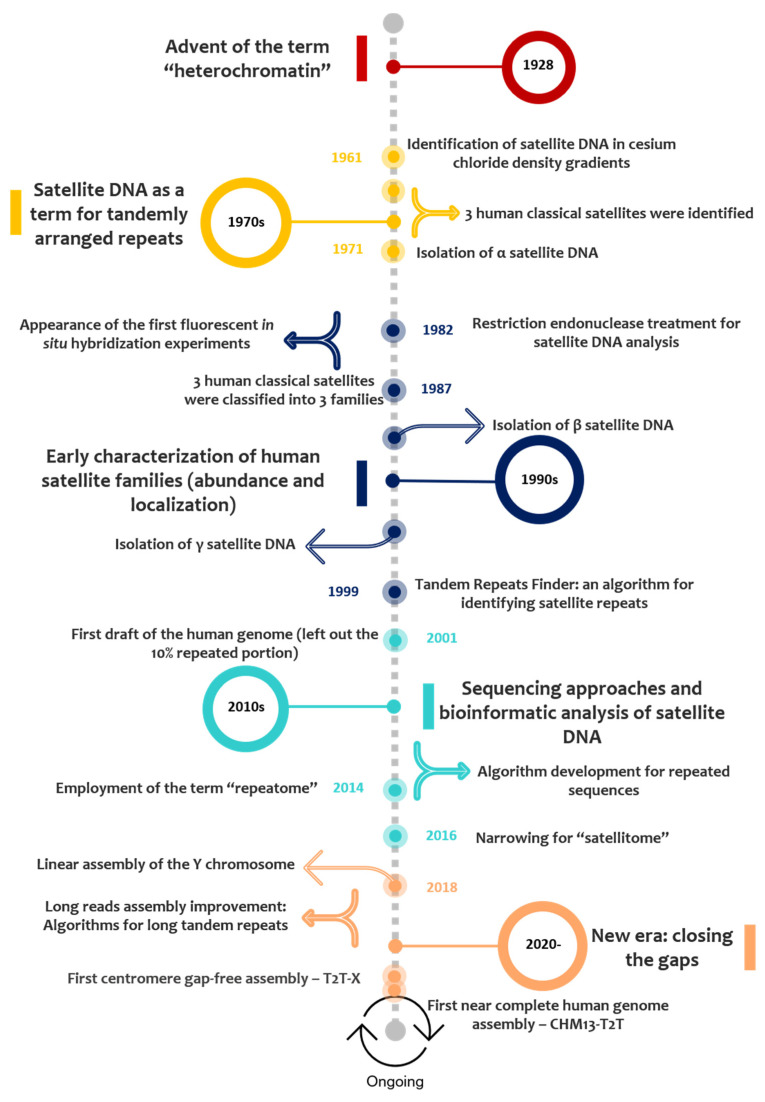
Landmarks in human satellite DNA research. The depicted timeline represents the knowledge evolution of satellite DNA biology in terms of classification, representativity, and significance, as well as the potential future and ongoing character of substantial breakthroughs in the area [7,9,10,29,30,31,45,46,51,52,53,58,73,74,75,79,87,92,96,107,117,120,127,128,150,162,173,184,185,186,187,188,190,191,192,193,194,195,196,197,198,199].

In the last fifty years, since the first efforts to characterize human satellite DNA families, a variety of research landmarks have spearheaded the development of the field of satDNA biology (Figure 2). The appearance of restriction enzyme treatment for satDNA analysis [184] or the development of in situ hybridization [185,186] supported the attainment of the first descriptions of the main human satellite families. With the progressive growth of sequencing technologies, several satDNAs have been sequenced and analyzed in specific and independent studies. However, they have somewhat been left aside from genome assemblies, essentially due to the previously discussed technological constrains of short-read sequencing. Nonetheless, in more recent years, the emergence of terms like ‘‘repeatome’’ [187] or ‘‘satellitome’’ [188] highlights the advantages of gathering total information about satDNA diversity using sequencing data. In turn, long-read technologies have produced the possibility of finally assembling satellite sequences. Human X [120] and Y [96] chromosomes were the first targeted for a linear centromeric assembly with characterization of centromeric array data (α HOR units DXZ1 and DYZ3, respectively). Sequencing of the DYZ3 centromeric array relied on the use of a previously developed satDNA BAC library spanning the entire region, which is considered a laborious and possibly bias-prone process. On the other hand, sequencing and polishing of DXZ1 relied on the presence of unique markers [114]. Both historical landmarks set a high-quality precedent for the potential of centromere genomic studies. Twenty years after the first draft of the human genome, the T2T (responsible for fully sequencing the X chromosome) just released a new human genome assembly for all chromosomes using the CHM13 haploid cell line, excitingly bringing the first wide-ranging glance of (peri)centromeric and acrocentric short arm regions. This project provides a near-completed assembly of the human genome, as only 5 rDNA-related gaps remain. Thus, high-resolution analysis excitingly became a reality for highly repetitive genomic regions [106,124].

Additionally, HiCanu software development [150], aimed at quality improvement for pre-existing reads, has resulted in linear assembly predictions spanning centromeric regions of chromosomes 2, 3, 7, 8, 10, 12, 16, 19, and 20, an achievement that clearly demonstrates the importance of upgrading assembler software. Beyond that, the linear assembly of chromosome 7 allowed one to confirm the presence of other repetitive elements like pericentromeric satellite families [114]. Bioinformatic tools can also be applied to determine HOR structure and variability, allowing one to distinguish apparently shared satDNA repeats, especially between acrocentric chromosomes [28]. Unraveling (peri)centromeric structural diversity will eventually allow us to expand our knowledge about the evolution of these type of sequences [71].

In the previous section, we emphasized the advances of technique interdependency, for example in the form of cytogenetically-assisted assembly. Along this line, future genomic innovation will probably rely on the combination of the maximum number of informative techniques. Ideally, high-quality array characterization should extend from *αSAT* repeats to other satellite families, primarily by exploring a broad inventory covering the variability of satDNA (sub)families. Despite clear efforts in representing centromere sequences in the human reference genome, all available human assemblies (previously to T2T consortium) drastically underrepresent *SATII*/*SATIII* repeats [39], a gap that has not even been acknowledged for the remaining families. Nevertheless, this type of underestimations caused by the collapse of identical repeats can be avoided by using raw read information for each satellite family [41,67]. Henceforth, it is essential to genomically update/confirm the available information about satDNA (sub)families, as several questions are unavoidable: number of (sub)families, genomic representativity, biological significance, and overall existence. Through the analysis of the CHM13 genome assembly from T2T, the meticulous analysis of satDNA (sub)families is finally a near reality.

Since human satDNA families present such a complex variability between individuals, and even between homologous chromosomes, the next genomic challenges will probably be centered on exploiting these differences: upscaling linear assembly to diploid chromosomes by optimizing phased assembly [114]; and extending the concept of ‘‘reference’’ genome to a multidimensional pan-genome approach, fully representing human populational haplogroup variation [71]. This last goal is being currently addressed by the Human Pangenome Reference Consortium, which recently launched 64 assembled human haplotypes from 32 genomes uncovering previously uncharted human variation [189]. Essentially, it is clear that satDNA analysis can benefit from the most representative survey of human satellite variants and their possible functional significance [39].

## 5. Concluding Remarks

There is no doubt that satDNA has established a past, present, and future in breaking barriers. Some of the largest have been recently overcome with the breakthrough of telomere-to-telomere assembly. Still, the detailed characterization of human satDNA families must walk a long path. To achieve better centromere contiguity and overall knowledge of (peri)centromeric/acrocentric short arm regions, it is vital to bet on the thorough tactic of technique interdependency. Better than a flawless, versatile, and stand-alone technique, a combined approach, where one technique’s disadvantages are counteracted by another, should be used for attaining accuracy.

In this review, we tried to trail the route of obstacles in satDNA analysis and to draw attention to the disparities in the amount of available information for different families. Previous attitudes towards repetitive sequences, combined with the subsequent awareness of their biological importance, establish a clear dichotomy, alerting one to the yet uncharted world of human satDNA diversity. Thus, by broadening our satDNA knowledge through time, it is highly likely that we will yet encounter new forms of functionality.

## Figures and Tables

**Table 1:** Summary of currently recognized human satDNA families features. Different satDNA families present distinct traits and can be divided in AT-rich or GC-rich satellites [29,33,41,47,74,87,88,89,94,95,98,99]. 1-*SATII* presents large blocks on chromosomes 1 and 16. 2-*SATIII* is widely represented on chromosome 9 [45].

	Repeat Unit Size	Identified Subfamilies	HOR Formation	Chromosomal Presence	Genome Representativity	
*αSAT*	171 bp	SFs; 28 identified (e.g., pTRA-1/2/4/7)	✓	All	3–5%	AT-rich
*SATI*	42 bp	pTRI-6	✓	3; 4; All acrocentric	0.12%	
*SATII*	5 bp	3 mentioned, no name identified	✓	1^1^; 2; 5; 7; 10; 13–17; 21; 22	1.5% (together w/ *SATIII*)	GC-rich
*SATIII*	5 bp	pTRS-47; pTRS-63;	✓	Y; 1; 3–5; 7; 9^2^; 10; 13–18 ;20–22	1.5% (together w/ *SATII*)	
		pTR9-s3; pTRS-2;				
		pE-1/2; pR-1/2/4;				
		pK-1; pW-1				
*βSAT*	68 bp	pB3/4	✓	Y; 1; 3; 9; 19; All acrocentric	0.02%	
*γSAT*	220 bp	GSAT; GSATX; GSATII	**-**	All	0.13%	

## Data Availability

Not applicable.
